# Analysis of Antioxidant Capacity of Chromones in *Saposhnikoviae Radix* Obtained by Ultrasonic-Assisted Deep Eutectic Solvents Extraction

**DOI:** 10.1155/2020/8875788

**Published:** 2020-12-28

**Authors:** Xianwen Yue, Fangfei Xu, Peng Lv, Huailei Yang, Huiwei Bao, Yang Xu

**Affiliations:** ^1^College of Pharmacy, Baicheng Medical College, Baicheng 137000, China; ^2^Plant Chemistry Laboratory, Chinese Institute of Jilin Ginseng, Changchun 130033, China; ^3^Pharmaceutical Department, The Second Hospital of Jilin University, Changchun 130000, China; ^4^College of Pharmacy, Changchun University of Chinese Medicine, Changchun 130117, China; ^5^School of Basic Medical Sciences, Jilin University, Changchun 130021, China

## Abstract

In this paper, ultrasonic-assisted deep eutectic solvent (DES) extraction was applied to the acquisition of chromones (cimicifugin, prim-o-glucosylcimifugin, and 5-o-methylvisamminoside) from *Saposhnikoviae radix* (SR). The extraction effects of 11 prepared DESs were screened taking contents of chromones as indexes. Furthermore, the optimum extraction conditions were confirmed using a single-factor test and response surface optimization test. Scavenging activities of DPPH anion and ABTS cation radicals of different SR extracts (DES, methanol, and ethanol) were studied. The analysis results of best extraction conditions optimized by Design-Expert software were as follows: extraction time (40 min), extraction temperature (60°C), and the solid/liquid ratio (32 mL/g). Scavenging rates of the DES extract for DPPH anion radical and ABTS cation radical were found to be 75.31% and 65.71%, which were higher than those of methanol and ethanol extracts. In conclusion, the developed extraction method can be regarded as a safe, green, and more effective approach for the extraction of chromones in SR.

## 1. Introduction

*Saposhnikoviae radix* (SR) is derived from the dry roots of *Saposhnikovia divaricata* (Turcz.) Schischk without flower and stem. The flavor of SR is pungent and slightly sweet, and the nature is warm. SR has the effects of inducing diaphoresis to dispel wind, relieving convulsion, and overcoming dampness [1]. SR is a traditional Chinese medicine with a long history, which was listed as the top grade in the “Herbal Classic of Shennong.” SR is mainly used for the treatment of exogenous wind-cold, aches all over the body, headache, dizziness, and damp retention [2].

SR contains chromones, coumarins, organic acids, polysaccharides, polyacetylenes, and sterols components in which chromones are the main active constituents [3–6]. According to research, prim-o-glucosylcimifugin (POG) and 5-o-methylvisamminoside (5OM) can not only decrease the temperature of febrile rats but also has obvious antipyretic, analgesic, anti-inflammatory, and antiplatelet aggregation effects [7]. Moreover, the content of cimicifugin (CIM) is found to be the highest in plasma. Since glycosides in SR (including POG and 5OM) must be converted into CIM in the intestine before being absorbed [8]. Therefore, CIM, POG, and 5OM, the three most representative chromones, were selected as the index components for the assessment of extraction effects of SR.

Research on SR is relatively comprehensive in recent years, including chemical composition, pharmacological effects, identification, and relevant quality standards [9]. A review of the studies on SR indicates that all in-depth research relies on the active components. Thus, it is of vital importance to increase the extraction efficiency of active ingredients.

The concept of deep eutectic solvents (DESs) was proposed for the first time in 2003 [10]. DES is a rapidly emerging, new, and green solvent, which can be used to replace traditional solvents and ionic liquids. DES is composed of two or more hydrogen bond donors and hydrogen bond acceptors in a certain molar ratio. It has the advantages of low melting point, low cost, low toxicity, easy preparation, regeneration, and biodegradability [11]. In addition, DES possesses excellent physical and chemical properties, such as adjustable viscosity, wide range of polarity, and good solubility. Consequently, DES has a broad application prospect in the separation, extraction, and synthesis of food and pharmaceutical chemical fields. At present, a large amount of literature on the application of DES in the separation and extraction of traditional Chinese medicine (TCM) has been reported [12–15].

In this paper, ultrasound-assisted DES method was applied to the extraction of chromones in SR in order to optimize the composition of DES. The optimum proportion of DES, ultrasonic extraction time, extraction temperature, and ultrasound power were investigated using a single-factor experiment. Antioxidant capacity of chromones extracted from SR was studied by evaluation of scavenging rates for DPPH anion and ABTS cation free radicals.

## 2. Materials and Methods

### 2.1. Materials and Reagents

**Figure 1:**
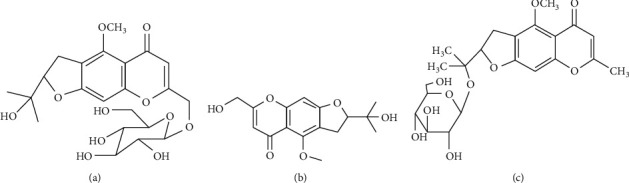
Chemical structure of chromones in SR: (a) POG; (b) CIM; (c) 5OM.

SR was obtained from Jilin Baiqi Co., Ltd. (Baicheng, China) and was identified by associate professor Xianwen Yue of Pharmacy College of Baicheng Medical College. CIM (batch number: 111710-200602), POG (batch number: 111522-201913), and 5OM (batch number: 111523-201811) were purchased from China Pharmaceutical Biological Products Verification Institute (Beijing, China). Methanol (Fisher, America) was of chromatographic grade. Phosphoric acid (excellent purity) was obtained from Guangfu Technology Development Co., Ltd. (Tianjin, China). Purified water was purchased from Hangzhou Wahaha Co., Ltd. Choline chloride, maltose, L-alanine, PL-malic acid, betaine, lactic acid, fructose, phenol, glycerol, propylene glycol, xylitol, urea, acetic acid, and citric acid were all acquired from Zhengzhou Kangyuan chemical products Co., Ltd (Henan, China). 1,1-Diphenyl-3-nitrophenylhydrazine (DPPH) was bought from Ruji Biotechnology Co., Ltd. (Shanghai, China). 2,2′-Azino-bis(3-ethylbenzothiazoline-6-sulfonic acid)diammonium salt (ABTS) and potassium persulfate were obtained from Macklin Biochemical Co., Ltd. (Shanghai, China). The chemical structure of three most representative chromones in SR is shown in Figure 1.

### 2.2. Instrumentations

Chromatographic analysis was performed on Agilent 1260 high-performance liquid chromatography (HPLC) system (including quaternary low-pressure mixing pump, autosampler, column oven, diode array detector, and chemstation workstation). AB135-S electronic balance was purchased from Mettler Toledo International Co., Ltd. KQ-250 ultrasonic cleaner was obtained from Kunshan Ultrasonic Instrument Co., Ltd. 78-1 magnetic heating stirrer was acquired from Changzhou Guohua Appliance Co., Ltd. Q-901 vortex mixer was bought from Haimen Qilinbeier Instrument Manufacturing Co., Ltd.

### 2.3. Chromatographic Conditions

Determination was achieved on Alltima™ C_18_ column (250 mm × 4.6 mm, 5 *μ*m) along with the column temperature of 30°C. The mobile phase was composed of methanol (*A*) and 0.1% phosphoric acid solution (*B*). The flow rate was sustained at 1.0 mL/min. The detective wavelength was set as 254 nm. The injection volume of the sample was 10 *μ*L. The specific gradient elution conditions were as follows: 0∼10 min, 5% ⟶ 20%A; 10∼20 min, 20% ⟶ 55%A; 20∼35 min, 55% ⟶ 55%A; 35∼60 min, 55% ⟶ 100%A.

### 2.4. Preparation of Mixed Standard Solutions

CIM (6.4 mg) was weighted precisely and dissolved with methanol (final adjusted volume 5 mL) to obtain CIM standard solutions for use. POG (8.32 mg) and 5OM (6.84 mg) were weighted accurately, and CIM standard solutions (precise 1 mL) were placed in the same volumetric flask and dissolved with methanol (final adjusted volume 10 mL) to obtain mixed stock standard solutions. Finally, mixed stock standard solutions (precise 0.5 mL) were taken and dissolved with methanol (final adjusted volume 10 mL) to obtain mixed standard solutions with the concentration of CIM 6.4 *μ*g/mL, POG 41.6 *μ*g/mL, and 5OM 34.2 *μ*g/mL. The solutions above were filtered via a 0.22 *μ*m membrane filter before HPLC analysis.

### 2.5. DES Preparation

The DESs were prepared using the stirring heating method at a constant temperature. The selected components were accurately weighed according to the calculated value of the mole ratio and placed in 100 mL conical flasks. The DES was obtained by stirring at 100°C (20∼120 min) until a stable and homogeneous liquid was formed. The types of DES prepared in this paper are displayed in Table 1.

### 2.6. Extraction Procedures

50 mg of SR powder (granularity, ≤100 mesh) and 1.5 mL of the prepared DES were added into the 2 mL centrifugal tube with cap. Subsequently, the tube was placed in a 60°C water bath for 5 min and was shaken via vortex for 5 min, followed by ultrasound treatment (40 KHz and 250 W) for 30 min. Then, the centrifugal tube was centrifuged for 5 min at the speed of 3000 r/min after cooling. Finally, the supernatant (precise 0.2 mL) was taken and dissolved with methanol (final adjusted volume 1 mL) to obtain test solutions. The test solutions were filtered through a 0.22 *μ*m microporous membrane before HPLC analysis. In addition, methanol and ethanol were used as reference extraction solvents instead of DES to obtain reference test solutions based on the method above.

### 2.7. Determination of Antioxidant Capacity

#### 2.7.1. Scavenging Activity for DPPH Anion Radical

DPPH, 1,1-diphenyl-3-nitrophenylhydrazine, is a kind of stable free radicals with a nitrogen center. The principle of DPPH detection is to combine a stable radical in the DPPH molecule with an electron pair provided by antioxidants. The formed colorless combination products will lighten the typical purple of free radical solutions [16, 17]. Extraction solutions of SR with different concentrations (5.10, 4.25, 3.40, 2.55, 1.70, 1.02, and 0.51 mg/mL) and DPPH anionic solutions (50 *μ*g/mL) were prepared with methanol first. Then, extraction solutions with different concentrations (0.1 mL) and DPPH anionic solutions (0.1 mL) were mixed, shaken well, and placed in dark for 30 min to obtain DPPH test solutions. The absorbance of DPPH test solutions (*n* = 3) was analyzed at 517 nm. Scavenging rate of DPPH radical was calculated on the basis of the following equation using methanol as blank control:

(1) $$\begin{array}{c} \text{Scavenging rate of DPPH radical}=\frac{A_{0}-\left(A_{1}-A_{2}\right)}{A_{0}}\times 100\%, \end{array}$$

where *A*_0_: absorbance of test solutions (0.1 mL DPPH + 0.1 mL methanol), *A*_1_: absorbance of test solutions (0.1 mL DPPH + 0.1 mL extraction solutions), and *A*_2_: absorbance of test solutions (0.1 mL methanol + 0.1 mL extraction solutions).

#### 2.7.2. Scavenging Activity for ABTS Cation Radical

ABTS can be oxidized by active oxygen to form bluish-green ABTS cation radical. However, antioxidants can be reacted with ABTS cation radical to discolor the ABTS radical solution [18]. ABTS (0.038 g) and potassium persulfate (0.0075 g) were weighed accurately and dissolved with deionized water (final adjusted volume, 10 mL), respectively. Then, equal amount of these two solutions was mixed and reacted in a dark place for 12 h. The reacted solutions (1.0 mL) were taken and dissolved with methanol (final adjusted volume 10 mL) to obtain ABTS cation solutions. Extraction solutions of SR with different concentrations (5.10, 4.25, 3.40, 2.55, 1.70, 1.02, and 0.51 mg/mL) were prepared with methanol. Then, extraction solutions with different concentrations (0.05 mL) and ABTS cation solutions (0.15 mL) were mixed, shaken well, and placed in dark for 2 h to acquire ABTS test solutions. The absorbance of ABTS test solutions (*n* = 3) was determined at 734 nm. Scavenging rate of ABTS radical was calculated according to the following equation using methanol as blank control:

(2) $$\begin{array}{c} \text{Scavenging rate of ABTS radical}=\frac{A_{3}-\left(A_{4}-A_{5}\right)}{A_{3}}\times 100\%, \end{array}$$

where *A*_0_: absorbance of test solutions (0.15 mL ABTS + 0.05 mL methanol), *A*_1_: absorbance of test solutions (0.15 mL ABTS + 0.05 mL extraction solutions), and *A*_2_: absorbance of test solutions (0.15 mL methanol + 0.05 mL extraction solutions).

## 3. Results and Discussion

### 3.1. Selection of DES System for Extraction of Chromones in SR

#### 3.1.1. Effect of Different Types of DES

**Figure 2:**
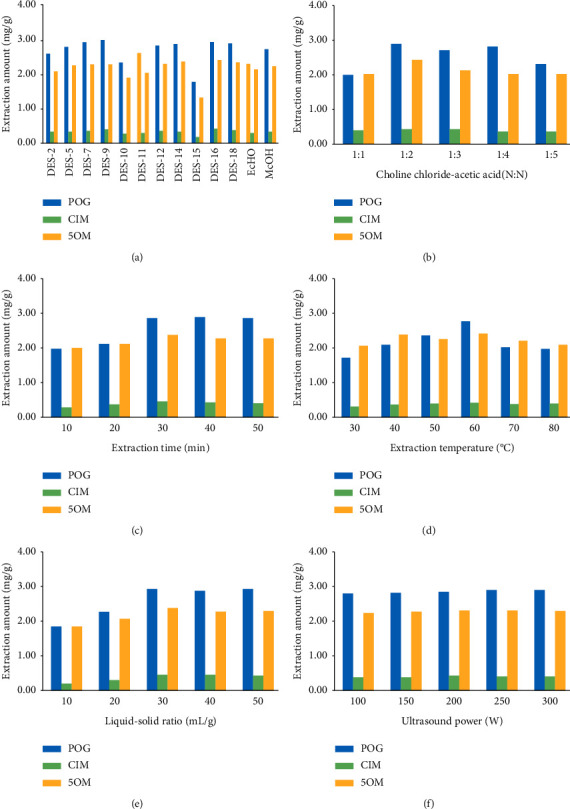
Effects of different types of DES (a), different ratios of DES (b), different extraction times (c), different extraction temperatures (d), different liquid/solid ratios (e), and different ultrasound powers (f) on the extraction rates of chromones.

**Figure 3:**
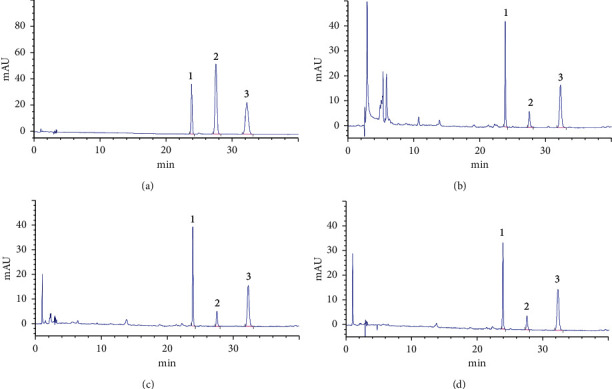
HPLC chromatograms of mixed standard solutions (a); DES test solutions (b); methanol test solutions (c); ethanol test solutions (d) (1: POG, 2: CIM, 3: 5OM).

The effect of different types of DES on the content of chromones in SR is shown in Figure 2(a). The HPLC chromatograms of CIM, POG, and 5OM in mixed standard solutions and test solutions prepared with DES composed of choline chloride-acetic acid are displayed in Figure 3. Some DES cannot be regarded as extraction solvents due to their solid or viscous liquid status. Thus, a certain proportion of water should be added to increase the fluidity of extraction solvents and improve the extraction efficiency and feasibility [19, 20]. Extraction conditions were as follows: liquid/solid ratio (30 mL/g), ultrasound power (40 KHz, 250 W), extraction time (30 min), and extraction temperature (60°C). As shown in Figure 2(a), better extraction effects could be achieved when DES consisted of choline chloride-lactic acid, choline chloride-fructose-water, choline chloride-xylitol-water, and choline chloride-acetic acid, especially choline chloride-acetic acid. Contents of CIM, POG, and 5OM were 0.4259, 2.9195, and 2.4075 mg/g using choline chloride-acetic acid as extraction solvents. However, contents of CIM, POG, and 5OM were 0.3030, 2.2908, and 2.1374 mg/g using methanol as extraction solvents. Contents of CIM, POG, and 5OM were 0.3455, 2.7206, and 2.2329 mg/g using ethanol as extraction solvents. The extraction efficiency of DES (choline chloride-acetic acid) was better than that of conventional solvents. Therefore, DES composed of choline chloride-acetic acid was selected as the optimum DES type.

#### 3.1.2. Effect of Different Ratios of Choline Chloride-Acetic Acid

Different ratios of choline chloride-acetic acid (N : N; 1 : 1, 1 : 2, 1 : 3, 1 : 4, and 1 : 5) were selected and prepared into DES used for the extraction of chromones in SR. Extraction conditions were as follows: DES (choline chloride-acetic acid), liquid/solid ratio (30 mL/g), ultrasound power (40 KHz, 250 W), extraction time (30 min), and extraction temperature (60°C). The content changes of CIM, POG, and 5OM in SR extracted by DES with different ratios of choline chloride-acetic acid are exhibited in Figure 2(b). The best composition of DES was choline chloride-acetic acid (N : N; 1 : 2) in the light of the extraction rate of chromones.

#### 3.1.3. Effect of Extraction Time

SR was extracted at different times including 10, 20, 30, 40, and 50 min to optimize the extraction conditions. Extraction conditions were as follows: DES (choline chloride-acetic acid, 1 : 2), liquid/solid ratio (30 mL/g), ultrasound power (40 KHz and 250 W), and extraction temperature (60°C). As displayed in Figure 2(c), the contents of chromones increased along with the growth of extraction time. However, the extraction rate reached a constant value after 30 min. The active components cannot be extracted completely within a short time usually. The stability of the substance, especially the activity of DES, will be affected by long time extraction. Moreover, the extraction efficiency will be influenced by long-time extraction as well [21, 22]. As a result, 30 min was considered as the best extraction time.

#### 3.1.4. Effect of Extraction Temperature

Influence of different extraction temperature (30, 40, 50, 60, 70, and 80°C) on extraction rates was investigated in order to extract the target compounds to the maximum extent. Extraction conditions were as follows: DES (choline chloride-acetic acid: 1 : 2), liquid/solid ratio (30 mL/g), ultrasound power (40 KHz, 250 W), and extraction time (30 min). As can be seen in Figure 2(d), the extraction rate of chromones in SR was higher when the extraction temperature was set as 60°C. This may be due to the fact that when extraction temperature increases, the internal temperature of the medicinal materials increases as well, which will promote the dissolution of the active ingredients. However, the active components will be destroyed under excessive temperature. Finally, 60°C was confirmed as the appropriate extraction temperature.

#### 3.1.5. Effect of Liquid/Solid Ratio

The liquid/solid ratio is one of the most important factors affecting the extraction efficiency. However, the extraction efficiency does not always increase along with the liquid/solid ratio. The appropriate proportion of liquid/solid can not only maximize the extraction of target compounds but also reduce the use of solvents and save the extraction cost [23]. In this study, different liquid/solid ratios of 10, 20, 30, 40, and 50 mL/g were inspected to screen the best extraction conditions. Extraction conditions were as follows: DES (choline chloride-acetic acid: 1 : 2), ultrasound power (40 KHz, 250 W), extraction time (30 min), and extraction temperature (60°C). As can be observed in Figure 2(e), the contents of chromones extracted from SR began to decrease since the liquid/solid ratio of 30 mL/g. Consequently, the liquid/solid ratio of 30 mL/g was chosen for further study.

#### 3.1.6. Effect of Ultrasound Power

In recent years, a great deal of research has shown that ultrasonic extraction is one of the ideal methods to extract natural active ingredients. It not only has the characteristics of high efficiency, simple operation, and short time but also has obvious advantages in protecting the chemical structure and biological activity of active components [24, 25]. In this paper, ultrasound power (100 W, 150 W, 200 W, 250 W, and 300 W), an important parameter for ultrasonic extraction, was checked. Extraction conditions were as follows: DES (choline chloride-acetic acid: 1 : 2), liquid/solid ratio (30 mL/g), extraction time (30 min), and extraction temperature (60°C). As shown in Figure 2(f), there was little difference between extraction rates of five ultrasound powers. Ultrasound power had little influence on the extraction effects of chromones in SR.

### 3.2. Optimization Experiment of Response Surface

An optimization experiment of response surface was performed in order to obtain the best extraction process parameters of target compounds. Influence factors of ultrasonic-assisted extraction of chromones from SR with DES (choline chloride-acetic acid), extraction time (*A*), extraction temperature (*B*), and liquid/solid ratio (*C*) were investigated on the basis of single-factor experiment. The results are shown in Table 2.

The Design-Expert software was used for the multiple regression fitting of data in Table 2. The results are displayed in Table 3. The lack of fits of the three models was more than 0.05, and the correlation coefficient (*R*^2^) was more than 0.95. Besides, the three models were all significant (*P* < 0.01). The results suggested that these models were of high-fitting degrees. The equations could well reflect the relationship between each factor and response surface. The multiple regression equations of POG (*Y*_1_), CIM (*Y*_2_), and 5OM (*Y*_3_) on the independent variables including extraction time (*A*), extraction temperature (*B*), and liquid/solid ratio (*C*) were as follows:

(3) $$\begin{array}{c} Y_{1}\left(\text{mg}/\text{g}\right)=3.38+0.16A+0.4B+5.26E-03C+0.025AB+0.046AC-0.047BC-0.064A^{2}-0.41B^{2}-0.022C^{2},\\ Y_{2}\left(\text{mg}/\text{g}\right)=0.54+0.021A+0.05B-5.20E-03C-0.013AB+4.02E-03AC+0.011BC-0.018A^{2}-0.076B^{2}-0.021C^{2},\\ Y_{3}\left(\text{mg}/\text{g}\right)=3.22+0.074A-0.16B+0.044C-0.089AB-0.018AC-0.015BC-0.082A{}^{2}-0.16B{}^{2}-0.1\mathrm{C.}{}^{2} \end{array}$$

**Figure 4:**
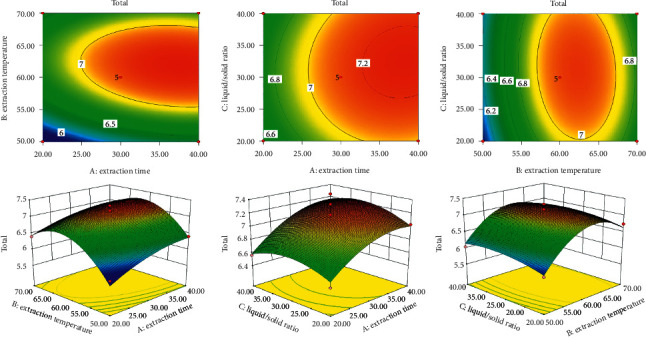
Response surface drawings of total chromones analyzed by interaction of two factors.

The surface of each factor (Figure 4) was relatively steep, indicating that extraction time, extraction temperature, and solid/liquid ratio had a significant influence on extraction effects. Figure 4 is analyzed according to the principle that the steeper the surface slope of response surface drawing and the denser the contour lines, the greater the interaction between the two factors [8, 26–28]. The results suggested that the curved surface slope of response surface formed between factor *B* (extraction temperature) and factor *C* (solid/liquid ratio) was the steepest among response surfaces of two factors interaction. The interaction between extraction temperature and solid/liquid ratio was the strongest.

Based on the analysis results of software, the best extraction conditions of SR were as follows: extraction time (40.00 min), extraction temperature (60.55°C), and the solid/liquid ratio (31.58 mL/g). The predicted value of contents of POG, CIM, and 5OM in chromones extracted by the above conditions was 3.5032, 0.5445, and 3.2005 mg/g. Taking the operability of the experiment into account, the optimized conditions were modified as follows: extraction time (40 min), extraction temperature (60°C), and the solid/liquid ratio (32 mL/g). The average contents of POG, CIM, and 5OM of three parallel validation tests were 3.4599, 0.5393, and 3.1304 mg/g, which was in accordance with the predicted value. The results showed that the optimized extraction conditions were stable and feasible.

### 3.3. Determination of Antioxidant Capacity

#### 3.3.1. Scavenging Activity for DPPH Anion Radical

**Figure 5:**
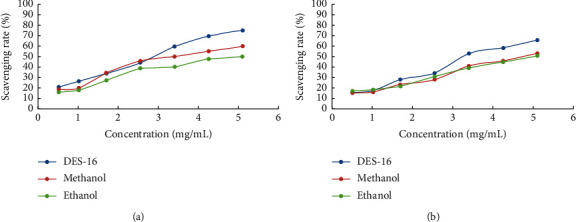
Scavenging rates of different concentrations of SR extracts on DPPH anion (a) and ABTS cation (b).

Scavenging rates of different concentrations of SR extracts on DPPH anion are displayed in Figure 5(a). The results suggested that scavenging rates of DES extracts (0.51 mg/mL and 5.1 mg/mL) on DPPH anion were 20.99% and 75.31%, respectively. The higher the concentration of SR extract solutions, the stronger the scavenging ability for DPPH anion. The scavenging rates of methanol and ethanol extracts (5.1 mg/mL) on DPPH anion were 59.93% and 50.33%, respectively. To sum up, the scavenging rate of SR extracted with DES (choline chloride-acetic acid) on DPPH anion was higher than those extracted with methanol and ethanol.

#### 3.3.2. Scavenging Activity for ABTS Cation Radical

Scavenging rates of different concentrations of SR extracts on ABTS cation are shown in Figure 5(b). The results indicated that scavenging rates of DES extracts (0.51 mg/mL and 5.1 mg/mL) on ABTS cation were 15.43% and 65.71%, respectively. The higher the concentration of SR extract solutions, the stronger the scavenging ability of ABTS cation. The scavenging rates of methanol and ethanol extracts (5.1 mg/mL) on ABTS cation were 53.18% and 50.52%, respectively. In a word, the scavenging rate of SR extracted with DES (choline chloride-acetic acid) on ABTS cation was higher than those extracted with methanol and ethanol.

## 4. Conclusions

In this paper, a green and new DES was used to extract the chromones from SR instead of traditional solvents. The optimal composition of DES was confirmed as choline chloride-acetic acid (N/N, 1 : 2). The optimum extraction conditions of DES were extraction time (40 min), extraction temperature (60°C), and the solid/liquid ratio (32 mL/g). The average contents of POG, CIM, and 5OM in chromones extracted by DES were 3.4599, 0.5393, and 3.1304 mg/g. Scavenging rates of DES extract for DPPH anion radical and ABTS cation radical were found to be 75.31% and 65.71%, which was higher than those of methanol and ethanol extracts. This study provides a new, efficient, and eco-friendly approach for extraction of chromones, which can be regarded as a promising, safe, and recommendable extraction strategy for extraction of active ingredients in the plant.

## Figures and Tables

**Table 1:** The types of prepared DES.

No.	Component (1)	Component (2)	N : N	H_2_O	Status (normal temperature)
DES-1	Choline chloride	Maltose	1 : 1	—	Viscous liquid
DES-2	Choline chloride	Maltose	1 : 1	30%	Transparent liquid
DES-3	Choline chloride	L-Alanine	1 : 1	—	Solid
DES-4	Choline chloride	PL-Malic acid	1 : 1	—	Viscous liquid
DES-5	Choline chloride	PL-Malic acid	1 : 1	30%	Transparent liquid
DES-6	Choline chloride	Betaine	1 : 1	—	Solid
DES-7	Choline chloride	Lactic acid	1 : 2	—	Transparent liquid
DES-8	Choline chloride	Fructose	1 : 1	—	Viscous liquid
DES-9	Choline chloride	Fructose	1 : 1	30%	Transparent liquid
DES-10	Choline chloride	Phenol	1 : 3	—	Transparent liquid
DES-11	Choline chloride	Glycerol	1 : 2	—	Transparent liquid
DES-12	Choline chloride	Propylene glycol	1 : 2	—	Transparent liquid
DES-13	Choline chloride	Xylitol	1 : 1	—	Viscous liquid
DES-14	Choline chloride	Xylitol	1 : 1	30%	Transparent liquid
DES-15	Choline chloride	Urea	1 : 2	—	Transparent liquid
DES-16	Choline chloride	Acetic acid	1 : 2	—	Transparent liquid
DES-17	Choline chloride	Citric acid	1 : 2	—	Viscous liquid
DES-18	Choline chloride	Citric acid	1 : 2	30%	Transparent liquid

**Table 2:** Design and results of response surface experiment.

No.	Factor (*A*)	Factor (*B*)	Factor (*C*)	Response results			
	Extraction time (min)	Extraction temperature (°C)	Liquid/solid ratio (mL/g)	POM (mg/g)	CIM (mg/g)	5OM (mg/g)	Total (mg/g)
1	20	60	20	3.3123	0.5494	3.2338	7.0955
2	30	60	30	3.3942	0.5403	3.0975	7.0320
3	30	70	40	3.0928	0.4535	3.0123	6.5586
4	30	60	30	3.3462	0.5302	3.2272	7.1036
5	40	60	40	2.4444	0.3729	3.0204	5.8377
6	40	60	20	3.4690	0.4810	2.7944	6.7444
7	30	60	30	3.0979	0.4891	2.8863	6.4733
8	30	60	30	3.0862	0.4919	2.8187	6.3968
9	30	50	40	2.5060	0.3819	3.1500	6.0379
10	40	70	30	3.2304	0.5479	3.1955	6.9738
11	20	60	40	3.5717	0.5207	3.1513	7.2437
12	30	50	20	3.5895	0.5355	3.2067	7.3317
13	30	70	20	3.4018	0.4918	2.7608	6.6544
14	30	60	30	3.3099	0.5095	2.8514	6.6708
15	40	50	30	3.3967	0.5365	3.2435	7.1767
16	20	70	30	2.6601	0.4256	3.3167	6.4024
17	20	50	30	2.4772	0.3969	3.0350	5.9091

**Table 3:** ANOVA for response surface quadratic model analysis of variance table.

Source	POG			CIM			5OM		
	Mean square	*F* value	*P* value	Mean square	*F* value	*P* value	Mean square	*F* value	*P* value
Model	0.25	15.62	0.0008	0.01	28.24	0.0001	0.06	43.04	<0.0001
*A*	0.21	13.30	0.0082	3.65*E* – 03	17.07	0.0044	0.04	33.36	0.0007
*B*	1.26	78.77	<0.0001	0.02	92.02	<0.0001	0.21	161.92	<0.0001
*C*	2.22*E* − 04	0.01	0.9097	2.16*E* − 04	1.01	0.3481	0.02	11.93	0.0106
*AB*	2.50*E* − 03	0.16	0.7050	6.97*E* − 04	3.26	0.1140	0.03	24.17	0.0017
*AC*	0.01	0.52	0.4943	6.47*E* − 05	0.30	0.5995	1.30*E* − 03	1.00	0.3500
*BC*	0.01	0.55	0.4823	4.72*E* − 04	2.21	0.1810	8.41*E* − 04	0.65	0.4472
*A* ^2^	0.02	1.08	0.3333	1.34*E* − 03	6.28	0.0406	0.03	21.56	0.0024
*B* ^2^	0.72	44.72	0.0003	0.02	115.05	<0.0001	0.11	83.73	<0.0001
*C* ^2^	1.99*E* − 03	0.12	0.7351	1.88*E* − 03	8.77	0.0211	0.04	34.39	0.0006
Lack of fit	0.01	0.74	0.5804	4.06*E* − 04	5.80	0.0613	0.01	3	2.51*E* − 03
*R* ^2^	0.9526	—	—	0.9732	—	—	0.9822	—	—
Adj. *R*^2^	0.8916	—	—	0.9387	—	—	0.9594	—	—

## Data Availability

The data used to support the findings of this study are included within the article.
